# Losartan and isoproterenol promote alterations in the local renin-angiotensin system of rat salivary glands

**DOI:** 10.1371/journal.pone.0217030

**Published:** 2019-05-22

**Authors:** Isadora Prado Cano, Thiago José Dionisio, Tânia Mary Cestari, Adriana Maria Calvo, Bella Luna Colombini-Ishikiriama, Flávio Augusto Cardoso Faria, Walter Luiz Siqueira, Carlos Ferreira Santos

**Affiliations:** 1 Department of Prosthodontics and Periodontology, Bauru School of Dentistry, University of São Paulo, Bauru, São Paulo, Brazil; 2 Department of Biological Sciences, Bauru School of Dentistry, University of São Paulo, Bauru, São Paulo, Brazil; 3 Department of Biochemistry and School of Dentistry, Schulich School of Medicine & Dentistry, The University of Western Ontario, London, Canada; Max Delbruck Centrum fur Molekulare Medizin Berlin Buch, GERMANY

## Abstract

Renin-angiotensin system (RAS) systemically or locally collaborates with tissue homeostasis, growth and development, which has been extensively studied for its pharmacological implications. This study was primarily aimed at finding and characterizing local RAS in rat parotid, sublingual and submandibular glands. It was also hypothesized that vasoactive drugs could affect the expression of RAS targets, as well as saliva flow and its composition. Therefore, another objective of this study was to compare the effects of losartan (angiotensin II receptor blocker) and isoproterenol (β-adrenergic receptor agonist). Forty-one Wistar rats were divided into three groups and administered a daily intraperitoneal dose of saline, losartan or isoproterenol solutions for one week. The following RAS targets were studied using qPCR: renin (REN), angiotensinogen (AGT), angiotensin converting enzyme (ACE), ACE-2, elastase-2 (ELA-2), AT1-a and MAS receptors, using RPL-13 as a reference gene. Morphology of glands was analyzed by immunohistochemistry using REN, ACE, ACE-2, AT1, AT2 and MAS antibodies. The volume and total protein content of saliva were measured. Our results revealed that ACE, ACE-2, AT1-a, AT2 and MAS receptors were expressed in all salivary gland samples, but REN and ELA-2 were absent. Losartan decreased mRNA expression of RAS targets in parotid (MAS) and submandibular glands (ACE and both AT receptors), without affecting morphological alterations, and significantly decreased saliva and total protein secretions. Isoproterenol treatment affected gene expression profiles in parotid (ACE, ACE-2, AT1-a, MAS, AGT), and submandibular (ACE, AT2, AGT) glands, thus promoting acinar hypertrophy in serous acini, without significant changes in salivary flow or total protein content. These drugs affected mainly acini, followed by duct systems and myoepithelial cells, whereas blood vessels were not affected. In conclusion, there is a local RAS in major rat salivary glands and losartan, an angiotensin II receptor blocker, affected not only the RAS-target gene expression but also decreased salivary flow and total protein content.

## Introduction

The description of the Renin-angiotensin system (RAS) would allow an improved understanding of homeostatic regulatory mechanisms, involving primarily vasodilation, water intake and sodium balance. The RAS classically works through the following cascade: renin (REN, originated from the juxtaglomerular cells of the kidney) cleaves angiotensinogen (AGT, a protein produced by the liver) into angiotensin-1 (Ang I: Asp^1^-Arg^2^-Val^3^-Tyr^4^-Ile^5^-His^6^-Pro^7^-Phe^8^-His^9^-Leu^10^). Angiotensin converting enzyme (ACE, obtained from lungs) then cleaves the Phe^8^-His^9^ bond to produce the vasoactive octapeptide angiotensin-2 (Ang II: Asp^1^-Arg^2^-Val^3^-Tyr^4^-Ile^5^-His^6^-Pro^7^-Phe^8^). Ang II binds to angiotensin type 1 receptors (AT1) leading to vasoconstriction, aldosterone secretion, fibrosis, proliferation, oxidative stress and inflammation among others, which have been described previously [1,2].

Other components also influence these reactions through different mechanisms [3]. For example, angiotensin-1-7 (Ang 1–7: Asp^1^-Arg^2^-Val^3^-Tyr^4^-Ile^5^-His^6^-Pro^7^) binds to MAS receptors (MAS) which has vasodilatation, antiproliferative, antithrombotic and antifibrotic effects [3,4]. In fact, 37 gene products have been described as an extended RAS [3].

Apart from the circulatory system, RAS also contributes to systemic development, which seems to affect bone healing [5] and the immune system [6]. It is also known that RAS cascade components can be locally synthesized in some other tissues [3], such as gingiva [7], adipose tissue [8], and brain [9]; the latter exhibits controversial functions [10]. Studies indicate that there could be local secretion in glands, which is regulated by the tissue’s RAS, as shown in mice lacrimal glands [11], as well as in rat, mice and human adrenal glands [12,13].

RAS cascade is affected when the systemic blood pressure is unbalanced; therefore, antagonists or inhibitors of RAS components are usually prescribed to re-establish normal systemic conditions. The most prevalent treatments of chronic hypertension comprise of ACE inhibitors (ramipril, captopril, enalapril, fosinopril, lisinopril and quinapril) [14] and/or angiotensin receptor (AT) blockers (losartan, candesartan, eprosartan, irbesartan, telmisartan and valsartan) [15], which were shown to have comparable antihypertensive effects.

Selective blockades of RAS may affect tissue function; Losartan impairs aldosterone production by the adrenal cortex [12], while valsartan increases mean tear production in lacrimal glands [16] and captopril up-regulates thirst sensation and water intake [17]. The influence of hypertension on salivary flow has been reported [18]. It is known that hypertensive and non-treated rat models also exhibit impaired saliva flow [19] with s quantitative and qualitative decrease in protein expression [20] and impaired local blood flow [18]. Since Ang-II-treated rats also present with an increase in water and sodium intake [21], RAS sensitivity of local vascular receptors could imply on a specific control of exocrine secretions as well as a direct role in salt and water retention [18].

Another condition is acute hypertension (or acute stress), which is known to trigger the release of adrenaline and noradrenaline, that bind to adrenergic receptors, resulting in many effects, such as increase in heart rate, peripheral paling or flushing, release of energy sources, mydriasis and inhibition of lacrimal and salivary gland secretions, among others [22].

Therefore, adrenergic receptors are related to saliva secretion, depending not only on signals from the central nervous system (CNS) but also from the circulatory system [23,24]. In summary, vasoactive peptides and neuro signs cause muscarinic and α-adrenergic stimulation to provide the fluid component of saliva. Afterward, β-adrenergic receptors coordinate with the release of specific proteins for each type of salivary glands [24]. Isoproterenol (β-adrenergic agonist) can reduce saliva release and increase the release of specific proteins due to a specific adrenergic stimulation [25,26]. Although not prescribed for clinical usage [26], this drug is used by researchers to understand stress adaptive mechanisms [25,27] and the mechanism through which it induces RAS components to coordinate with tissue fibrosis, proliferation and protection, in the heart [28] and kidney [27].

In the context of salivary glands microscopic anatomy, it is important to note that, in humans, parotid gland has a serous profile and is, responsible for majority of the stimulated saliva flow, while the sublingual gland is predominantly mucous, and the submandibular gland behaves as a mixed gland responsible for 60% of the unstimulated saliva secretion. While similar features are detected in human parotid and sublingual glands as those in rodents, rat submandibular glands are characterized by an exclusive serous profile that shows a major potential to release growth factors from granular ducts [29]. Granular convoluted tubules (GCT) are a unique characteristic of rodent submandibular glands and are able to release granules with a variety of biologically active polypeptides, with local and systemic functions [29]. The differences between species should be considered for further studies using animal models to explain tissue physiology without the technical and bioethical limitations that apply to experiments on human tissues.

The absence of saliva synthesis impairs quality of life and protection against oral diseases [25,29]. Xerostomia could be a complaint in healthy patients although it is more often considered to be a consequence of chronic diseases or a collateral effect of their treatments, such as hypertension [17,20], cardiovascular diseases, hyperthyroidism and diabetes mellitus [30]. Thus, the dry mouth has become a common complaint on a regular clinical basis.

Given the oral impact that RAS could have in coordinating with local homeostasis, the hypothesis of the present study was that RAS components are synthesized in the salivary glands and that pharmacological prescription of an AT1 receptor antagonist and a β-adrenergic receptor agonist modulates RAS expression and salivary secretion. Therefore, the present study aimed at investigating RAS components produced locally in rat parotid, submandibular and sublingual glands, to characterize whether or not they were local RAS or not. Secondly, pharmacological approaches were used to promote two distinct alterations: an AT blocker (losartan) was prescribed to elucidate local alterations caused by a RAS target blockade, whereas a β-adrenergic agonist (isoproterenol) was taken as a positive control on the basis of previous reports on alterations in salivary glands function and morphology [31,32]. Finally, in order to evaluate the impact of such drugs in physiology and saliva secretion, the saliva volume and total protein measurements were performed.

## Materials and methods

All the methods were carried out under the guidelines of the National Research Council. All the animal protocols were approved by the Ethics Committee for Animal Experiments of Bauru School of Dentistry, University of São Paulo (#015/2013).

### Preparation of animals

Forty-one 60-day-old male Wistar rats, weighing between 248–339 grams were obtained for experiments from the Animal Breeding Center of the Bauru School of Dentistry, University of São Paulo, and housed in temperature-controlled rooms, with free access to food and water. The rats were divided into three groups, each receiving one specific pharmacological treatment for a week, consisting of a daily intraperitoneal dose of saline (negative control group, n = 12), losartan (10 mg/kg body weight (b.w.) [33], n = 14) or isoproterenol (20 mg/kg b.w. [31,32], n = 15). Three rats from isoproterenol group died before the seventh day (S1 and S3 Tables), and this could be expected since isoproterenol treatment is also used to induce experimental chronic heart failure in rat models [34] and myocardial infarction [35] with different doses.

### Immunohistochemistry and quantitative gene expression

**Fig 1:**
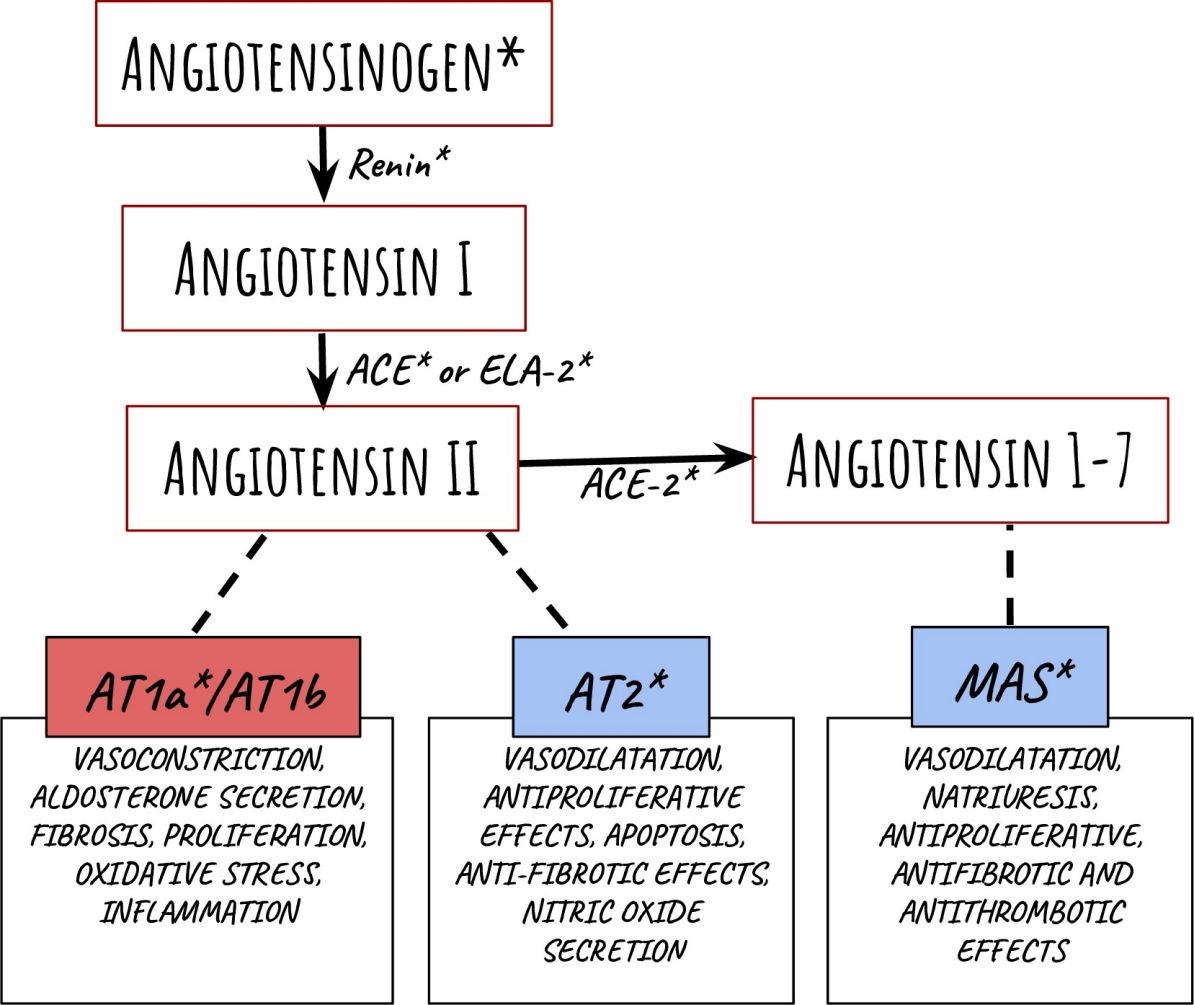
Representation of the metabolic RAS cascade. Angiotensinogen and the peptide sequences are shown as a result of specific enzyme cleavages, which are included next to their represented arrows. The receptors are represented as boxes on the bottom and the function described is a result of the linked angiotensin binding. The RAS targets used in this study are indicated with an asterisk. Graphic illustration is adapted from Ferrario and Strawn [1].

For histological and qPCR analyses (targets are represented in Fig 1), 21 rats were used (for each treatment group n = 7; by the last day of the experiment, 2 rats from isoproterenol group died, being the only group with n = 5). Basal salivary secretion was inhibited by subcutaneous injection of 0.5 mg/Kg b.w. of atropine sulfate (1.25% atropine solution, Prado Laboratory SA, Brazil) on the last day, for all experiments, in order to revoke the sialogogue effect caused by the drugs used for euthanasia [36]. It is important to mention that, to the best of our knowledge, the effect of atropine in the expression of the local RAS in salivary glands is not yet described and, in the present experiment, it affected all animals equally. After 10 minutes, the animals were anesthetized by an intraperitoneal injection of ketamine chloride (55 mg/kg b.w.) and xylazine chloride (10 mg/kg b.w.). Immediately, parotid, submandibular and sublingual glands pairs were collected and divided into two groups. For the first group, the glands were harvested, maintained in microcentrifuge tubes containing a solution for RNA stabilization (RNAlater, Ambion, USA), and stored in the freezer at -80°C until RNA extraction. The second group (contralateral gland) was fixed in 10% neutral buffered formaldehyde (pH 7.2) for 24 hours and processed using routine histological techniques for immunohistochemical analysis. The animals were euthanized with excessive anesthetic dose, after gland harvesting.

#### Total RNA isolation, cDNA synthesis and quantitative RT-PCR

Total RNA was isolated from each gland using the RNeasy Mini Kit (Qiagen, Valencia, USA), according to the manufacturer’s instructions, after weighing and separating 20–30 mg tissues. RNA quantity and quality were measured using a spectrophotometer (NanoDrop 1000, Thermo Scientific). Quanti Tect Reverse Transcription Kit (Qiagen, Valencia, USA) was used to perform reverse transcription (RT) according to the manufacturer’s instructions. The contents of the kit were mixed with RNA samples, followed by incubation of the reaction mixture at 37°C for 30 minutes in a Veriti Thermal Cycler (Applied Biosystems, USA). Transcriptase was inactivated post transcription by incubating the samples at 95°C for 3 minutes. Transcripted samples with cDNAs were stored in the freezer (-20°C).

cDNAs were mixed with the Taqman gene expression master mix (Applied Biosystems, USA) and gene specific TaqMan primer/probe sets (Table 1). A pilot study in our group did not find expression of AT1b receptors in sublingual and submandibular glands (unpublished). Therefore, this study focused on only the AT1-a and AT2 receptors (Fig 1).

**Table 1:** Primers used for quantitative gene expression.

Target	Catalog number
**AGT**	Rn00593114_m1
**REN**	Rn00561847_m1
**ACE**	Rn00561096_m1
**ACE-2**	Rn01416289_m1
**AT1-a**	Rn00578456_m1
**AT2**	Rn00560677_s1
**MAS**	Rn00562673_s1
**ELA-2**	Rn00561147_m1
**RPL-13**	Rn00821258_g1

AGT, angiotensinogen; REN, renin; ACE, angiotensin converting enzyme; AT, angiotensinogen II receptor; MAS, MAS receptor; ELA, elastase. The primers are recommended by the qPCR kit manufacturer (Applied Biosystems, USA).

#### Gene expression data analysis

Relative gene expression was quantified based on RPL13 as the reference gene, by subtracting the target’s threshold cycle (Ct) from RPL13’s threshold cycle to obtain delta CT (ΔCt). Applied Biosystems assures 100% efficiency in the reactions using their kits since these assays have been previously validated by the company. Therefore, relative quantification (qPCR) was based on the following formula 1+ reaction efficiency−^ΔΔCt^ (2^– ΔΔCt^). Statistical analysis was performed with One-way ANOVA/ Tukey post-test for parametric results, whereas Kruskal-Wallis/ Dunn’s post-test was applied for non-parametric results. Differences were considered to be statistically significant at *p* < 0.05.

### Immunohistochemistry

REN, ACE, ACE-2, AT1, AT2 and MAS were selected as targets for immunohistochemical analysis. The glands were embedded in paraffin enriched with polymers (Histosec^TM^, Merck). Four-micron thick sections were obtained and mounted on silane-coated glass slides (Dako S 3003). The paraffin sections were deparaffinized, re-hydrated and immersed in deionized water for subsequent immunohistochemical procedures. Primary antibodies used for specific staining of the RAS targets are listed in Table 2.

**Table 2:** Origin, isotype, concentration and dilution of the primary antibodies used for the immunohistochemical staining.

Target	Catalog N#	Isotype	Concentration	Dilution*
**REN**	Sc 27318	IgG goat	200 μg/mL	1/75
**ACE**	ab 11734	IgG mouse	200 μg/mL	1/75
**ACE-2**	Sc 20998	IgG rabbit	200 μg/mL	1/50
**AT1**	Sc 1173	IgG rabbit	200 μg/mL	1/50
**AT2**	ab 19134	IgG rabbit	200 μg/mL	1/50
**MAS**	Sc 390453	IgG mouse	200 μg/mL	1/75

RAS targets (SantaCruz Biotechnology): REN, renin; ACE, angiotensin converting enzyme; AT, angiotensinogen II receptor; MAS, MAS receptor; ELA, elastase.

*antibody diluent (ADS-125, Spring Bioscience Corp, Pleasanton, California, USA).

Slides were immersed in epitope retrieval buffer (10 mM sodium citrate and 0.05% Tween 20 at pH 6.0) in a pressure cooker at 15 psi/121°C for 5 min. Endogenous peroxidase activity was blocked for 10 min with hydrogen peroxide block (DHP-125, Spring Bioscience Corp) and the serum proteins in a 7% milk solution (Molico^®^, Nestlé, Brazil) for 15 minutes. Tissue sections were incubated in each primary antibody as specified in Table 2 for 1 hour in a wet chamber. Slices were carefully rinsed in Tris-buffered saline (TBS) and incubated in Universal Immuno-enzyme Polymer combining amino acid polymers with peroxidase and the Fab' fragment of the secondary antibody (N-Histofine^®^ Simple StainTM Rat MAX PO, Nichirei Biosciences INC., Japan). Rat kidney was similarly processed and used as positive and negative controls. Antibody diluent was used instead of the primary antibody for negative control. Sections were treated with 3,3'-diaminobenzidine tetrahydrochloride (DAB-125, Spring Bioscience Corp) for 3 minutes and counterstained with Mayer’s hematoxylin for 3 minutes, followed by viewing under the microscope. Immunolabeling patterns of the RAS targets in the glands were determined using a light microscope (Axioskop, Carl Zeiss, Germany) and photomicrographs were taken with a high-resolution digital camera (AXIOCAM HRc; Carl Zeiss) using a 40x oil immersion objective lens.

### Salivary flow and total protein measurements

Nineteen rats (5 treated with saline, 7 treated with losartan and 7 treated with isoproterenol) were used for salivary flow and total protein measurements the day after the last proposed drug administration, as described previously by Benarde [37]and Bighetti [38]. Saliva was collected during 15 minutes after the first drop noticed following pilocarpine injection. Total volume was calculated from the difference in weight between empty and saliva containing Falcon® 50 mL tubes, considering saliva density as 1 mg/mL. The volume measurement was further confirmed with precise pipetting (μL) of the samples. As noticed, rat body weight influenced saliva secretion, therefore, the salivary flow rate was normalized and expressed as μL/min/g body weight. For total protein measurements, the Bradford method was used. Both saliva volume and saliva total protein tests were parametric, therefore data were expressed as means ± standard deviation and analyzed by One-way ANOVA test/Tukey post-test. Differences were considered to be statistically significant at *p* < 0.05.

## Results

### Quantitative gene expression

All the glands used in this study did not express the targets of REN and ELA-2; however, the expression of the targets ACE, ACE-2, AGT, AT1-a, AT2 and MAS was detected in all the samples with characteristic profiles in each gland. Comparison between the treatment groups showed statistical differences.

#### Parotid gland

**Fig 2:**
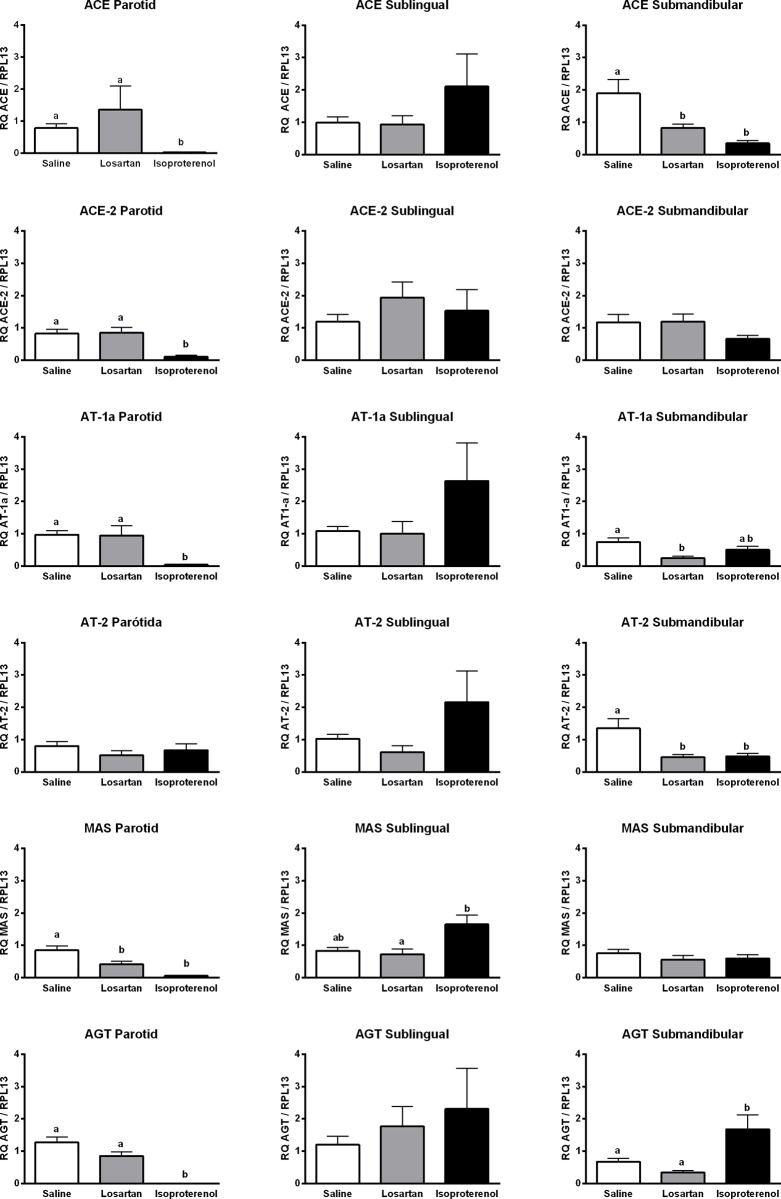
Relative quantification of each target in rat parotid, sublingual and submandibular glands. Data are means ± standard deviation. One-way ANOVA/Tukey’s multiple comparison tests were performed for parametric results whereas Kruskal-Wallis/ Dunn’s post-test was applied for non-parametric results. Differences were considered statistically significant when p<0.05. Different letters indicate a statistically significant difference between groups and when such differences were calculated between two groups but not in a third one as compared to both, the latter is represented as ab. Letters were not used in the graph when differences were not significant between saline, losartan and isoproterenol.

Our data indicate that losartan treatment decreased the MAS expression, but the other targets showed no statistical differences when compared with saline as control (Fig 2). Isoproterenol reduced the expression of the majority of the targets with statistically significant differences for ACE, ACE-2, AT1-a, MAS and AGT.

#### Sublingual gland

The sublingual gland was the least affected among all the glands studied, since slight but no significant statistical differences between both treatments were observed when compared with saline.

#### Submandibular gland

The submandibular gland was the most affected by both treatments, amongst all the other glands. While losartan decreased the expression of ACE, AT1-a and AT2, whereas isoproterenol decreased the expression of AT2 and ACE and increased the expression of AGT (Fig 2).

### Immunohistochemistry

#### Negative and positive controls

**Fig 3:**
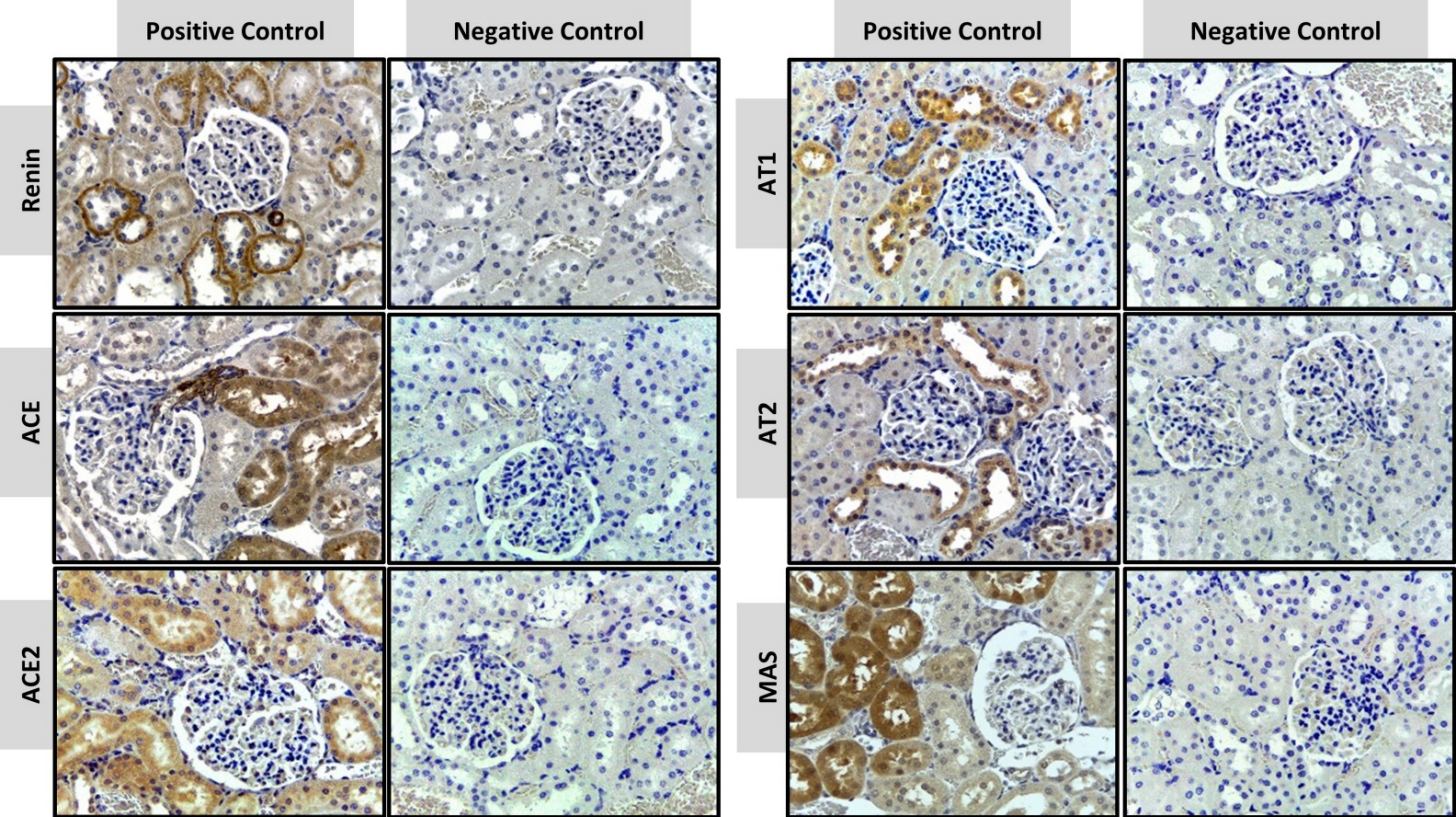
Positive and negative controls in immunohistochemical staining of RAS targets in the granular cortex of the kidney. Immunostains are shown as a brown color in positive controls of REN, ACE, ACE-2, AT1, AT2 and MAS. (x40 objective and scale bar = 50 μm).

The positive anatomical control used in this study was the kidney because the presence of RAS components in the kidney is known *a priori* [3], and it is not the target of the present experimental treatment. Fig 3 shows the RAS targets in the granular cortex, with the characteristic expression of each target. REN expression was observed in the juxtaglomerular cells and tubular cells. ACE was detected in the peritubular capillaries, in the brush border of proximal tubules and the arterial adventitia, while ACE-2 was localized to the proximal and distal convoluted tubules. AT1 was detected in all tubular cells, epithelial cells of Bowman's capsule and vascular smooth-muscle cells, but AT2 was localized only in tubular cells. MAS receptor was mostly found within the proximal convoluted tubules and was absent in the glomerulus. No signal was observed in the negative control of a kidney section by omitting the primary antibody, thus showing the specificity of immunostaining of the targets.

#### Major salivary glands

**Fig 4:**
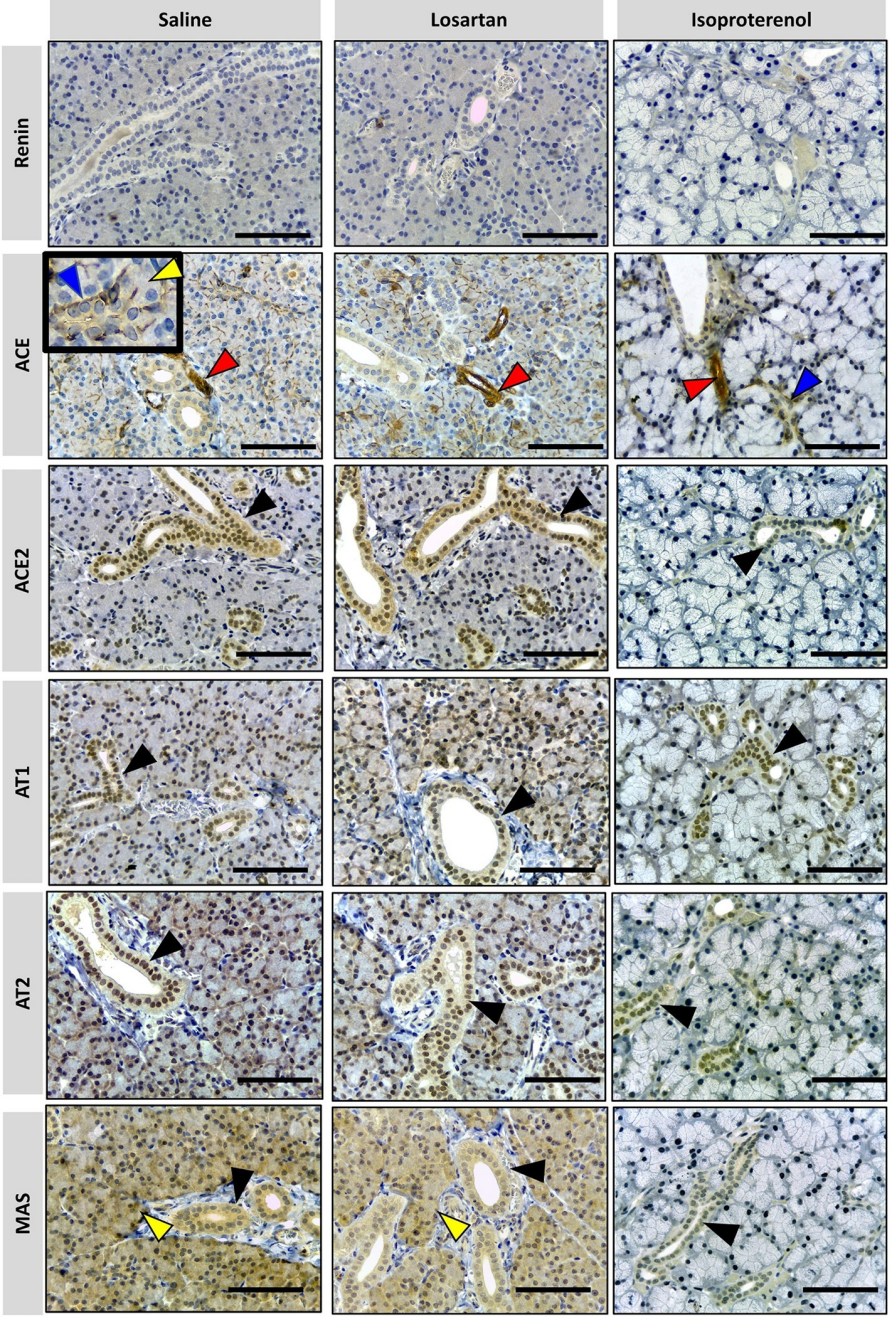
Photomicrographs of immunohistochemical staining (brown) of RAS targets in rat parotid gland. Targets: REN, ACE, ACE-2, AT1, AT2 and MAS were analyzed. Arrows indicated by colors: blue = ducts; yellow = acini; red = blood vessels; black = epithelial cells of the duct system. No REN expression in parotid glands was observed under all treatments. ACE is present in myoepithelial cells around intercalated ducts (indicated by blue arrowhead in detail), acini (indicated by yellow arrowhead in detail) and in blood vessels (indicated by red arrowhead). ACE-2, AT1 and AT2 are present in epithelial cells of the duct system (indicated by black arrowhead), while MAS is present in duct cells (black arrowhead) and acinar cells (blue arrowhead) in all treatments except for isoproterenol. Comparatively, the parotid glands of the animals which received saline and losartan show a similar pattern of immunolabeling for RAS targets but have more expression than in animals which received isoproterenol. (x40 objective and scale bar = 50 μm).

**Fig 5:**
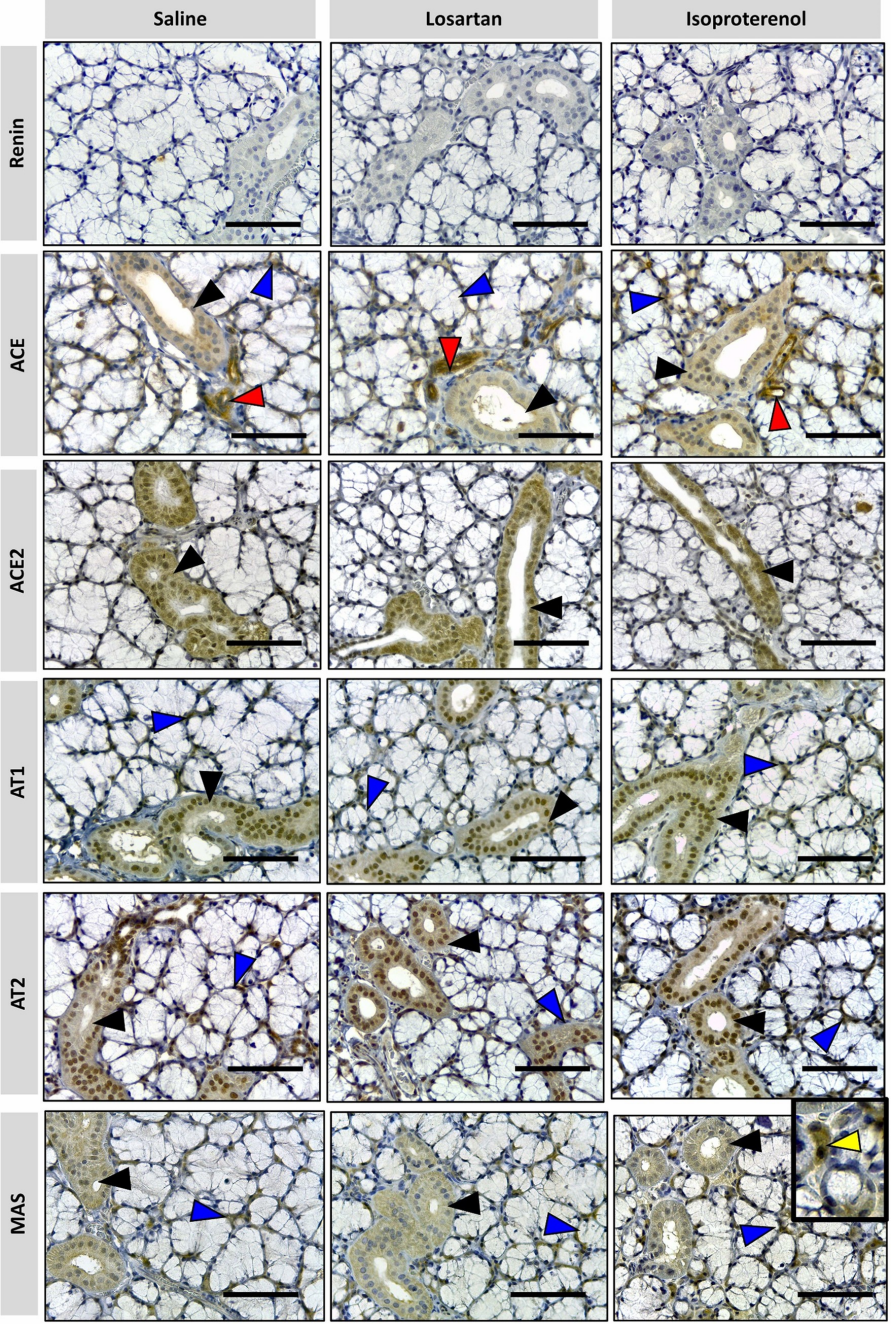
Photomicrographs of immunohistochemical staining (brown) of RAS targets in rat sublingual gland. Targets: REN, ACE, ACE-2, AT1, AT2 and MAS were analyzed. Arrows indicated by colors: blue = myoepithelial cell; black = duct cells; red: blood vessels; yellow: acinar cells. No REN expression was observed in sublingual glands in all treatments. ACE is present in myoepithelial (blue arrow), duct cells (black arrowhead) and blood vessels (red arrowhead). ACE-2, AT1, AT2, and MAS are present in epithelial cells of the duct system (black arrowhead), myoephitelial cells (blue arrowhead) and more rarely in serous acinar cells (yellow arrowhead) under isoproterenol treatment. Comparatively, the immunostaining patterns of the RAS targets are similar in the sublingual gland in all treatments. (x40 objective and scale bar = 50 μm).

**Fig 6:**
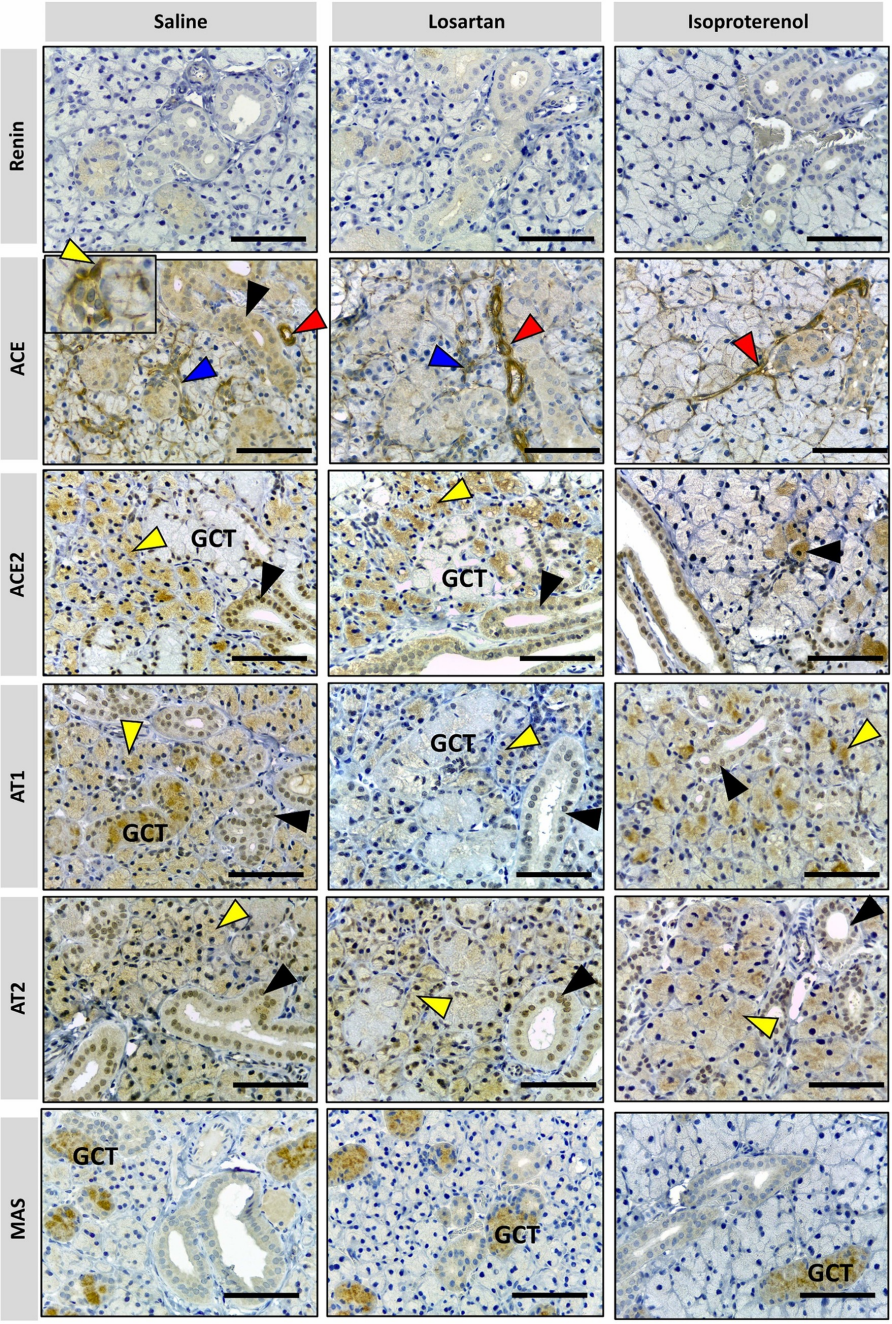
Photomicrographs of immunohistochemical staining (brown) of RAS in rat submandibular gland. Targets: REN, ACE, ACE-2, AT1, AT2 and MAS were analyzed. Arrow colors indicate as follows: blue = myoepithelial cell; black = duct cells; red = blood vessels; yellow: acinar cells. GCT = granular convoluted tubule. No REN expression was observed in submandibular glands under all treatments. ACE shows higher expression in blood vessels (red arrowhead) under all treatments. ACE expression in duct cells (black arrowhead) and myoepithelial cells around intercalated ducts (blue arrowhead) and acini (yellow arrowhead) are higher in saline treatment than those in rat glands treated with losartan and isoproterenol. ACE-2 is present in acinar cells (yellow arrowhead) and the duct system (black arrowhead) except in the granular convoluted tubule cells (GCT). Mild ACE-2 expression in acinar cells can be observed in rat glands treated with isoproterenol. AT1 and AT2 are present in acinar cells (yellow arrowhead) and striated/excretory ducts (black arrow head), but only AT1 is expressed in GCT. AT1 is weakly expressed in rat glands treated with losartan and a strong immunostain for MAS is observed only in the GCT. (x40 objective and scale bar = 50 μm).

RAS targets in three major salivary glands were detected by immunohistochemistry in formalin-paraffin sections. Figs 4–6 showed that the localization and intensity of immunostaining varied according to the type of gland and treatment.

The expression of RAS targets in salivary glands can be partially compared with mice lacrimal glands [39], especially when the epithelial cells of the duct systems are described. Also, myoepithelial cells were detected in the three studied glands and immunostained for RAS targets, thus suggesting a local function on duct contraction.

In contrast to previous reports of mice submandibular glands [40] and previously described by many authors [41,42], there is an absence of REN expression in the three salivary glands, in both treated and untreated rats. Therefore, the latter was also considered as a negative control in our procedure.

Parotid gland showed specific RAS targets marked in blood vessels, duct, and acinar cells. ACE was observed in myoepithelial cells and blood vessels. ACE-2, AT1, and AT2 were present in epithelial cells of the duct systems. MAS was present in the duct and acinar cells of the saline and losartan groups, being the only target significantly affected by this drug. Isoproterenol remarkably reduced RAS expressions in acinar cells but not in blood vessels and ducts, based on immunostaining analysis.

The sublingual gland naturally showed major expression of mucous cells with the only immunostains being observed in ducts, blood vessels and interstitial tissue. Besides REN, all the RAS targets were observed in epithelial cells of the duct systems and myoepithelial cells (the latter not being detected with ACE-2 immunomarkers). Therefore, no relevant alterations seem to be induced by either losartan or isoproterenol treatment groups in the sublingual gland.

In contrast to the other types of glands, the expression of RAS targets in the submandibular gland was present in acinar cells, and was not restricted to ducts only as in the case of the majority of the other glands. The GCT were heterogeneously stained, as clearly seen with MAS immunomarkers. Comparison of the different treatments showed that the expression of acinar cells was the most affected, as seen within losartan treatment for ACE, AT1 and AT2 groups. Based on qPCR results, isoproterenol decreased the expression of the targets AT2, ACE in acini. MAS expression remained restricted to GCT with an aspect of degranulated cytoplasm, in all the treatments, similar to the study reported by Thulesen et al. [43].

ACE was the only target identified in the blood vessels, responsible for the immunostaining of these cells for all salivary glands. It is important to note that none of the treatments impacted the expression of these targets.

### Salivary flow and total protein results

**Fig 7:**
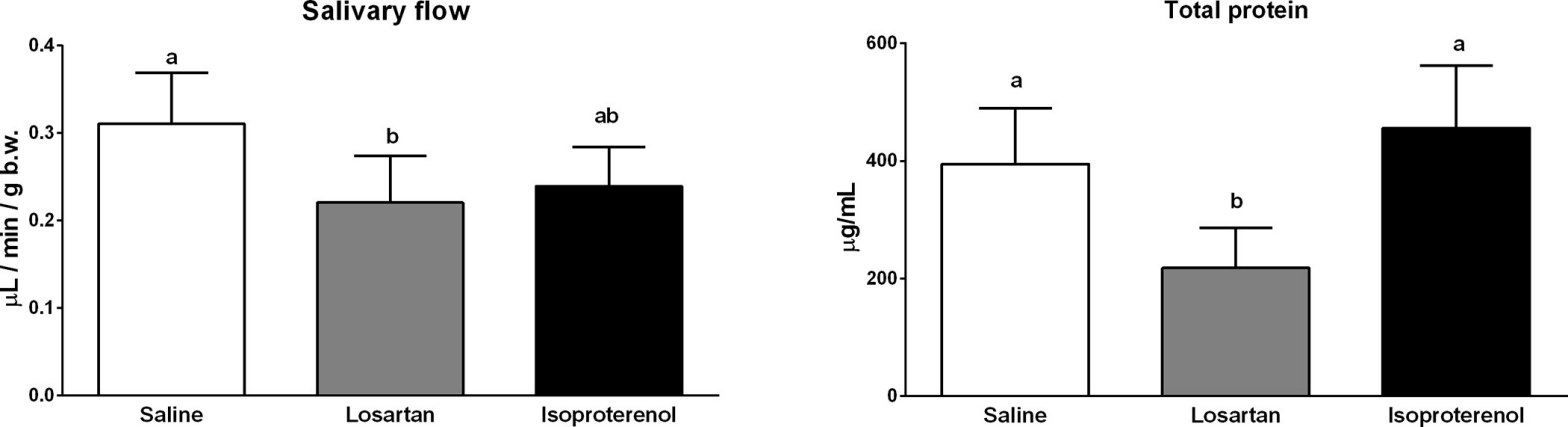
Salivary flow and total protein determination in control, losartan and isoproterenol groups. Data are presented as means ± standard deviation. Different letters indicate a statistically significant difference (p<0.05) using One-way ANOVA/Tukey’s multiple comparison test for salivary flow analysis and Kruskal-Wallis/Dunn test for total protein analysis.

Both methods for the of salivary flow (weighing tubes before and after saliva collection (S1 Fig) and pipetting the real volume in μL) resulted in similar data, statistical results, and analyzes. Therefore, the real volume comparison was presented here as it was considered a more accurate method of analysis. Total protein measurements are also represented in Fig 7.

Losartan reduced salivary flow and total protein secretion with a significant statistical difference when compared to saline (respectively, p = 0.0227 and 0.0415). However, no change occurs in gland morphologies based on immunohistochemistry and mass (S2 Table).

Consistent with other studies [31,32], isoproterenol induced morphological alterations and increased parotid and submandibular glandular mass (S2 Table). However, none of these results significantly affected salivary flow (p = 0.0771) or total protein release (p > 0.999) when compared with the control group. Notwithstanding, when compared with losartan, the salivary flow of isoproterenol-treated rats had no statistically significant difference (p = 0.773) and total protein release showed significantly higher results (p = 0.0037). Therefore, it can be concluded that isoproterenol promoted a saliva secretion with higher protein concentrations amongst the studied groups.

## Discussion

RAS components have been previously observed in salivary glands [41,44–48] and the present study proposes a possible explanation through which local RAS in salivary glands collaborates with saliva synthesis and tissue physiology. The vascular activity starts with local or systemic AGT cleavage, as observed from our qPCR analyses, which showed mRNA expression in all the glands studied. It is well known that after REN activity, ACE cleaves Ang I into Ang II in order to obtain this important vasoactive peptide.

As presented in our results and previously described [41,42], REN was not found in any of the analyzed glands (Figs 4–6). However, it has been shown in many species, including humans [49], that Ang II synthesis is not restricted to these enzymes. Cathepsin G, tonin and ELA-2 are enzymes responsible for alternative pathways in Ang II synthesis, replacing REN and/or ACE. In addition, local REN in rats could result from bloodstream uptake.

Opposing the absence of this enzyme in rats, different mice strains express REN in sublingual and mainly in submandibular glands, the latter noticed to be the main source of extrarenal REN in the majority of the studied mice [40]. This enzyme collaborates either locally, with hormonal and gender dependency, or systemically, regulating plasma concentration and blood pressure [50]. REN is an example of many targets with different expressions amongst other species, therefore it is important to bear in mind that the metabolism of rat salivary glands has its own characteristics when trying to prospect reasons for, i.e., human conditions.

Hence, when alternative enzymes were considered, none of the glands locally expressed ELA-2 mRNA, so this alternative RAS pathway for Ang I cleavage into Ang II [51] was discarded based on our results. Further investigations should target tonin, kallikreins and Cathepsin G in order to explain in further detail, how enzymes replace REN functions. There is evidence for tonin and kallikreins in submaxillary glands and their corresponding systemic REN levels, comparing normotensive and hypertensive rats [52]. Salivary glands are the main site for the gene expression of kallikreins, which are also responsible for balancing classical RAS with vasodilatation. They are located in convoluted tubes, intercalated ducts and in the main excretory duct cells [50], which is similar to the findings of this study with respect to many RAS targets. Therefore, RAS collaboration with homeostasis in these tissues could be complementary to the kallikrein-kinin axis.

This association with other vasoactive systems was previously discussed, when captopril caused dipsogenic effects in rats when bradykinin was studied as a stimulator of thirst [17]. ACE was predominantly detected in salivary myoepithelial cells, similar to lacrimal glands [39] and other exocrine glands [53], contouring acinar and ductal epithelial cells in a stellate format (ACE, blue arrow detail in Figs 4–6). They have both epithelial and smooth muscle cell features. Therefore, it is proposed that their contractions would induce the secretory function of these acini, as previously noticed in lacrimal glands [39]. Indeed, the characterization of myoepithelial cells presents novel perspectives on how salivary gland microtissues work, and therefore, proposes mechanisms for restoration of their function in hyposalivation [53].

Our results suggest that RAS mechanisms complying exclusively vasoconstriction and dilation are not able to completely describe salivary glands local mechanisms on saliva release, since the decrease of ACE expression in myoepithelial cells cannot explain lower salivary rates by itself. We propose that local ACE, in addition to other cascades triggered by AT1 blockade, would affect these cells contraction and further reduce saliva flow. It is partly proven since both treatments decreased saliva secretion (isoproterenol almost reached statistical significance with p = 0.0771) and had submandibular myoepithelial cells with attenuated ACE expressions.

Complementary, polydipsia was not a side-effect related to treatment with captopril [17]. The primary polydipsia associated with the inhibition of ACE declined inner medullary aquaporin (AQP) 2, without significant change AQP3 and AQP4 expression. Our results suggest, that RAS is involved in thirst regulation because AT1 blockade through losartan resulted in a decrease in ACE expression and salivary flow.

Concerning RAS receptors, Matsubara et al. [48] used binding assays for rat submandibular glands and observed that AT1 was predominant in comparison to AT2, although they authors did not include AT1 subtypes (AT1-a and AT1-b) which were used in our study.

As mentioned in the Materials and methods section, we found that AT1-b was present only in parotid glands in our preliminary experiment (unpublished data). Therefore, AT1-a subtype was specifically studied to compare all salivary glands. In contrast to the previous study [48], there were statistical differences between AT1 and AT2 expression levels in submandibular glands of the saline and losartan groups (Fig 2). Since isoproterenol did not reduce AT1 expression in submandibular glands when compared to saline, the hypothesis that RAS collaborates with cellular growth induced by this drug remains sustained [48]. However, it is not a sole cause since AT1 expression was inhibited by isoproterenol in parotid glands and hypertrophy was still noticed.

AT1 inhibition by losartan affected the submandibular gland gene expression exclusively, and GCT was the most affected cell group (Fig 6). Since total protein measurements decreased by this drug and GCT were important protein releasers, we suppose that the interaction of Ang II with AT1 exerts a controlling function of GCT cells. We assume that because AT1 was not equally affected by isoproterenol in GCT, and total protein in saliva was not decreased in the same manner as in losartan-treated rats.

qPCR data also revealed that AT2 expression was affected by losartan and GCT marks were attenuated. However, this alteration was not as remarkable as that for AT1 results. Since the literature is limited at describing RAS functions in salivary ducts, further studies should be undertaken to comprehend the function of both angiotensin receptors.

MAS receptors were observed in all the studied duct systems (Figs 4–6). In parotid gland, MAS expression was significantly decreased by both treatments, when compared to saline (Fig 1). In the sublingual gland, it was the only target which was increased by isoproterenol administration. Since this gland presents a mucous profile, it was expected to notice minor alterations on general protein expression. However, parotid and sublingual glands expressed MAS in ducts and acini, while the submandibular gland strictly expressed this target in GCT and presented no alterations when compared to saline.

Specific conditions were observed with respect to pharmacological alterations. Compared to saline, losartan did not affect glandular morphologies, although when parotid MAS and submandibular AT1, AT2 and ACE decreased, both salivary flow and total protein were affected. For instance, there is not a specific pathway able to explain RAS targets as direct participants of dilation or contraction of acini and ducts, as it is described for classical RAS in circulatory tissues (Fig 1).

Despite the alterations on macro and microscopic measurements [31,32], as well as affecting most of the RAS targets expression in parotid and submandibular glands, isoproterenol was not able to evoke significant alterations on salivary flow and total protein secretion, thus contradicting previous findings that reported an increased saliva output [43]. The expression of the targets was diminished in acinar cells; therefore, these results could be related to the maintenance of RAS expression in ducts. The impact of acinar alterations can be related to further variations in saliva composition during a time noticed when hyperstimulation affected salivary components, such as the Epidermal Growth Factor (EGF) synthesis and secretion in submandibular glands, incomplete peptide processing and depletion of cellular mature storages [43].

When Ang II is injected in rats, water and sodium intake increases [21] while submandibular gland blood flow decreases [18]. For the moment, the alterations provoked by AT receptors blockade are an increase of glandular blood flow [18] and potassium release in saliva [54]. Masajtis-Zagajewska et al. prescribed losartan for patients under dialysis to reduce thirst. In contrast to our results, losartan increased the stimulated salivary flow, however with no effects on xerostomia for treated patients. Therefore it was not recommended as an alternative to reduce water intake [54]. Since interdialytic weight gain was the same for treated and untreated patients, we assume that the water consumption was the same for both groups, and the amount of water intake would not be the cause of up or down regulation of saliva secretion induced by losartan. As rat and human results were contradictory when measuring saliva flow, more studies should be performed to confirm whether this is a species-related cause and has clinical relevance.

A mechanism of saliva inhibition that should be considered is that losartan enhances local blood flow in salivary glands but systemically reduces blood pressure [18]. Fazekas et al. [18] did not perform any salivary measurements, however since RAS blockade mildly affected local blood vessels receptors, the systemic circulation could be responsible for lower supplies for saliva synthesis.

In fact, each salivary gland demonstrates its own mechanism in self-modulation, as noticed when AGT expression was almost null in parotid glands treated with isoproterenol while it was significantly increased in submandibular glands of the same treatment.

To the best of our knowledge, the present study is the first to conclude that there is a local RAS in rat major salivary glands that collaborates with saliva synthesis and components. This could be assured since specific blockade of AT1 with losartan impaired saliva flow and total protein release, even without major alterations in macro and microscopic glandular features. In contrast, β-adrenergic stimulation with isoproterenol increased glandular mass and promoted cellular hypertrophy without causing significant differences in salivary and protein secretions.

## Supporting information

S1 Fig Salivary volume measured as the differences between tubes before and after saliva collection. The salivary flow rate was normalized and expressed as mL/min.100g/b.w, considering saliva density 1 mg/mL. Data are means ± standard deviation. One-way ANOVA/Tukey’s multiple comparison tests were performed. Differences were considered statistically significant when p<0.05. Different letters indicate a statistically significant difference between groups and, when such differences were noticed between two groups but not in a third one as compared to both, the latter is represented as ab.
(PDF)

S1 Table Rat weight (grams) after 7-day injection of saline, losartan and isoproterenol. Rats used for qPCR/immunohistochemistry analysis. * indicate rats in isoproterenol group that died before the experiment was finished.
(PDF)

S2 Table Samples weight (grams) of each group used for qPCR. Data are means ± standard deviation. One-way ANOVA/Tukey’s multiple comparison tests were performed. Differences were considered statistically significant when p<0.05. Different letters (a or b) indicate a statistically significant difference between groups, whereas equal letters indicate the absence of such differences. Letters were not used when differences were not significant between saline, losartan and isoproterenol. It is important to mention that the ipsilateral gland was sent for immunohistochemistry and is not included in the measurements of this table.
(PDF)

S3 Table Rat weight (grams) after 7-day injection of saline, losartan and isoproterenol. Rats were used for salivary flow measurements. * indicate rat in isoproterenol group that died before the experiment was ended.
(PDF)
