# Supplementary material for: Losartan and isoproterenol promote alterations in the local renin-angiotensin system of rat salivary glands
Source: PLoS One. 2019 May 22;14(5):e0217030. doi: 10.1371/journal.pone.0217030 (PMC6530859; doi:10.1371/journal.pone.0217030)
Supplement: S1 Fig — The salivary flow rate was normalized and expressed as mL/min.100g/b.w, considering saliva density 1 mg/mL. Data are means ± standard deviation. One-way ANOVA/Tukey’s multiple comparison tests were performed. Differences were considered statistically significant when p<0.05. Different letters indicate a statistically significant difference between groups and, when such differences were noticed between two groups but not in a third one as compared to both, the latter is represented as ab. (PDF) [file pone.0217030.s001.pdf]

S1 Fig. Salivary volume measured as the differences between tubes before and after saliva collection.

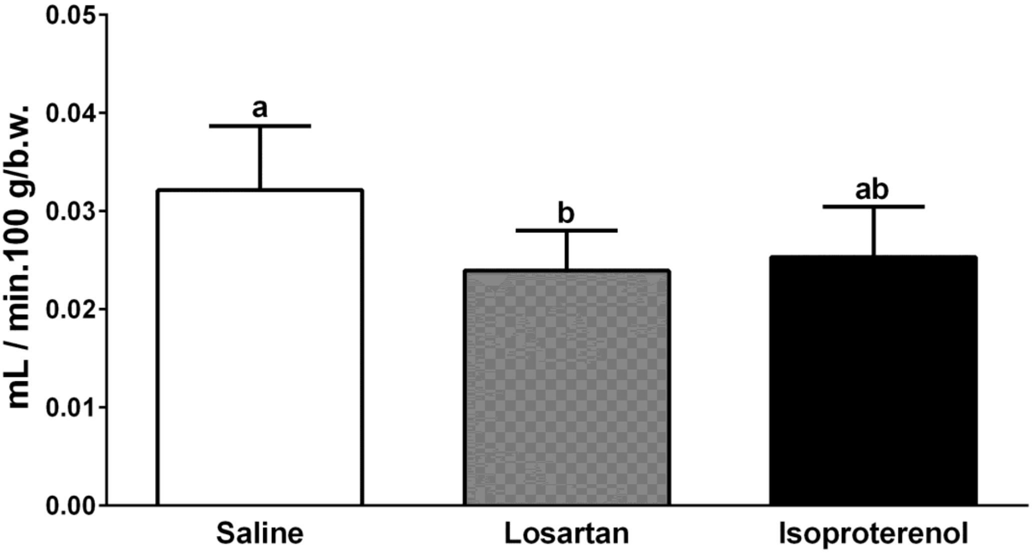

The salivary flow rate was normalized and expressed as mL/min.100g/b.w, considering saliva density 1 mg/mL. Data are means ± standard deviation. One-way ANOVA/Tukey's multiple comparison tests were performed. Differences were considered statistically significant when $p<0.05$. Different letters indicate a statistically significant difference between groups and, when such differences were noticed between two groups but not in a third one as compared to both, the latter is represented as ab.
