# Supplementary material for: Losartan and isoproterenol promote alterations in the local renin-angiotensin system of rat salivary glands
Source: PLoS One. 2019 May 22;14(5):e0217030. doi: 10.1371/journal.pone.0217030 (PMC6530859; doi:10.1371/journal.pone.0217030)
Supplement: S1 Table — Rats used for qPCR/immunohistochemistry analysis. * indicate rats in isoproterenol group that died before the experiment was finished. (PDF) [file pone.0217030.s002.pdf]

S1 Table. **Rat weight (grams) after 7-day injection of saline, losartan and isoproterenol.** * indicate rats in isoproterenol group that died before the experiment was finished.

SALINE	LOSARTAN	ISOPROTERENOL
312	332	335
319	303	307
248	300	323
324	357	304
336	324	330
315	341	*
352	355	*
