# Supplementary material for: Losartan and isoproterenol promote alterations in the local renin-angiotensin system of rat salivary glands
Source: PLoS One. 2019 May 22;14(5):e0217030. doi: 10.1371/journal.pone.0217030 (PMC6530859; doi:10.1371/journal.pone.0217030)
Supplement: S2 Table — Data are means ± standard deviation. One-way ANOVA/Tukey’s multiple comparison tests were performed. Differences were considered statistically significant when p<0.05. Different letters (a or b) indicate a statistically significant difference between groups, whereas equal letters indicate the absence of such differences. Letters were not used when differences were not significant between saline, losartan and isoproterenol. It is important to mention that the ipsilateral gland was sent for immunohistochemistry and is not included in the measurements of this table. (PDF) [file pone.0217030.s003.pdf]

S2 Table. Samples weight (grams) of each group destinated for qPCR.

	Parotid			Submandibular			Sublingual		
	**Saline**	**Losartan**	**Isoproterenol**	**Saline**	**Losartan**	**Isoproterenol**	**Saline**	**Losartan**	**Isoproterenol**
	217.5	221.4	394.7	153.8	169.2	232.7	38.4	39.1	34.3
	327.2	225.8	394.6	176.4	175.5	272.4	26.7	27.7	33.5
	235.5	185.1	475.3	159.7	162.1	342.2	35.8	27.7	29.8
	206.4	233	625.1	179.1	179.1	328.2	36.7	35.8	34.2
	265.6	175.1	551.5	147.8	161.0	325.9	35.7	39.3	43.8
	234.7	310.9	*	163.1	200.4	*	29	27.6	*
	267.5	216.1	*	183.1	183.7	*	44	34	*
**Mean**	**250.6[a]**	**223.9[a]**	**488.2[b]**	**166.1[a]**	**175.8[a]**	**300.2[b]**	**35.1**	**33.0**	**35.1**
**SD**	**40.6**	**43.9**	**100.5**	**13.5**	**13.7**	**46.1**	**5.7**	**5,30**	**5,10**

Data are means ± standard deviation. One-way ANOVA/Tukey's multiple comparison tests were performed. Differences were considered statistically significant when $p<0.05$. Different letters (a or b) indicate a statistically significant difference between groups, whereas equal letters indicate the absence of such differences. Letters were not used when differences were not significant between saline, losartan and isoproterenol. It is important to mention that the ipsilateral gland was sent for immunohistochemistry and is not included in the measurements of this table.
