# Supplementary material for: Losartan and isoproterenol promote alterations in the local renin-angiotensin system of rat salivary glands
Source: PLoS One. 2019 May 22;14(5):e0217030. doi: 10.1371/journal.pone.0217030 (PMC6530859; doi:10.1371/journal.pone.0217030)
Supplement: S3 Table — Rats were used for salivary flow measurements. * indicate rat in isoproterenol group that died before the experiment was ended. (PDF) [file pone.0217030.s004.pdf]

S3 Table. **Rat weight (grams) after 7-day injection of saline, losartan and isoproterenol.**

SALINE	LOSARTAN	ISOPROTERENOL
310	279	280
321	294	308
297	306	270
294	256	265
290	285	293
	251	282
	326	274
		*

Rats used for salivary flow measurements. * indicate rat in isoproterenol group that died before the experiment was finished.
